# Supplementary material for: Identification of Multiple Replication Stages and Origins in the Nucleopolyhedrovirus of Anticarsia gemmatalis
Source: Viruses. 2019 Jul 15;11(7):648. doi: 10.3390/v11070648 (PMC6669502; doi:10.3390/v11070648)
Supplement: Supplementary file 1 [file viruses-11-00648-s001.zip › Supplementary files/Table S1.docx]

**Table S1.** Putative DNA structures of Baculovirus ORIs.

| **Name** | **Number of structures** | **GB ID** | **Start (nt)** | **Length** | | | | | **AT%** | | | | | **End (nt)** |
| --- | --- | --- | --- | --- | --- | --- | --- | --- | --- | --- | --- | --- | --- | --- |
|  |  |  |  | **S1 (bp)** | **Linker 1**  **(nt)** | **S2**  **(bp)** | **Linker 2**  **(nt)** | **S3**  **(bp)** | **S1** | **Linker 1** | **S2** | **Linker 2** | **S3** |  |
| **AcMNPV *hr*1** | **3** | **KM667940.1** | **26** | **4** | **2** | **9** | **3** | **7** | **75** | **100** | **74.1** | **0** | **66.7** | **90** |
|  |  |  | **206** | **9** | **2** | **11** | **5** | **4** | **64.3** | **50** | **60** | **60** | **38.5** | **283** |
|  |  |  | **271** | **4** | **4** | **10** | **5** | **12** | **38.5** | **50** | **63.6** | **80** | **60** | **355** |
| **AcMNPV *hr*1a** | **1** |  | **7,748** | **4** | **3** | **6** | **5** | **4** | **81.8** | **66.7** | **73.7** | **80** | **83.3** | **7865** |
| **AcMNPV *hr*2** | **3** |  | **26,467** | **2** | **1** | **11** | **1** | **6** | **37.5** | **0** | **73.1** | **100** | **66.7** | **26,519** |
|  |  |  | **26,454** | **3** | **4** | **11** | **1** | **5** | **82.3** | **25** | **73.1** | **100** | **55.5** | **26,519** |
|  |  |  | **26,811** | **10** | **0** | **12** | **3** | **2** | **71.4** | **0** | **60** | **25** | **28.6** | **26,876** |
| **AcMNPV *hr*3** | **2** |  | **70,800** | **5** | **2** | **5** | **5** | **5** | **71.4** | **100** | **72.2** | **100** | **50** | **70,852** |
|  |  |  | **71,006** | **4** | **2** | **13** | **6** | **4** | **76.9** | **100** | **68.7** | **83.3** | **83.3** | **71,070** |
| **AcMNPV *hr*4b** | **2** |  | **97,633** | **5** | **3** | **9** | **3** | **5** | **78.9** | **66.6** | **78.6** | **33.3** | **66.6** | **97,703** |
|  |  |  | **97,710** | **5** | **0** | **11** | **2** | **12** | **76.9** | **0** | **64.5** | **50** | **60** | **97,782** |
| **AcMNPV *hr*5** | **2** |  | **117,623** | **3** | **5** | **12** | **3** | **4** | **38.4** | **60** | **63.3** | **33.3** | **61.5** | **117,686** |
|  |  |  | **117,674** | **4** | **3** | **5** | **4** | **3** | **61.5** | **33.3** | **86.6** | **25** | **70** | **117,718** |
| **AcMNPV**  **non-*hr*** | **6** |  | **114,002** | **5** | **2** | **7** | **6** | **3** | **33.3** | **100** | **45** | **80** | **36.4** | **114,057** |
|  |  |  | **114,301** | **4** | **1** | **11** | **6** | **4** | **61.5** | **100** | **70.6** | **66.7** | **75** | **114,366** |
|  |  |  | **114,385** | **3** | **4** | **6** | **4** | **4** | **60** | **50** | **35** | **75** | **63.2** | **114,441** |
|  |  |  | **114,652** | **5** | **5** | **5** | **2** | **4** | **66.7** | **80** | **64.7** | **50** | **52.4** | **114,713** |
|  |  |  | **114,760** | **5** | **2** | **9** | **2** | **2** | **36.4** | **50** | **65.8** | **100** | **57.1** | **114,840** |
|  |  |  | **115,099** | **3** | **5** | **7** | **5** | **4** | **27.3** | **80** | **30** | **40** | **31.2** | **115,155** |
| **LdMNPV *hr*1** | **1** | **AF081810.1** | **435** | **11** | **1** | **13** | **1** | **2** | **51.8** | **0** | **34.4** | **100** | **25** | **503** |
| **LdMNPV *hr*2** | **2** |  | **9,557** | **4** | **1** | **6** | **1** | **4** | **37.5** | **0** | **55.2** | **100** | **78.9** | **9,622** |
|  |  |  | **9,937** | **2** | **4** | **13** | **4** | **4** | **38.9** | **25** | **28.1** | **50** | **27.3** | **10,005** |
| **LdMNPV *hr*3a** | **1** |  | **30,087** | **3** | **4** | **11** | **1** | **5** | **37.5** | **37.5** | **28.1** | **100** | **35.7** | **30,087** |
| **LdMNPV *hr*3b** | **3** |  | **31,607** | **2** | **6** | **12** | **0** | **9** | **25** | **66.7** | **42.1** | **0** | **45.4** | **31,680** |
|  |  |  | **31,684** | **2** | **6** | **12** | **0** | **8** | **25** | **66.7** | **47.5** | **0** | **40.9** | **31,759** |
|  |  |  | **31,763** | **2** | **5** | **7** | **1** | **4** | **25** | **80** | **38.1** | **0** | **54.5** | **31,808** |
| **LdMNPV *hr*3c** | **1** |  | **33,452** | **9** | **4** | **9** | **3** | **3** | **39.3** | **50** | **57.1** | **100** | **15.4** | **33,533** |
| **LdMNPV *hr*4** | **2** |  | **46,089** | **9** | **2** | **13** | **5** | **11** | **40.9** | **0** | **47.5** | **60** | **34.4** | **46,189** |
|  |  |  | **46,265** | **4** | **3** | **11** | **2** | **10** | **33.3** | **50** | **50** | **0** | **44** | **46,344** |
| **LdMNPV *hr*5** | **2** |  | **88,459** | **4** | **1** | **9** | **2** | **3** | **7.7** | **0** | **50** | **0** | **20** | **88,506** |
|  |  |  | **88,786** | **7** | **6** | **10** | **0** | **2** | **61.5** | **66.7** | **32.1** | **0** | **25** | **88,853** |
| **LdMNPV *hr*6** | **1** |  | **119,930** | **7** | **0** | **6** | **5** | **8** | **44.4** | **0** | **45.8** | **0** | **55** | **119,996** |
| **LdMNPV *hr*7a** | **1** |  | **139,956** | **6** | **1** | **7** | **5** | **6** | **25** | **0** | **54.2** | **60** | **50** | **140,019** |
| **LdMNPV *hr*7d** | **1** |  | **148,780** | **9** | **1** | **13** | **5** | **5** | **44** | **0** | **35** | **80** | **41.2** | **148,867** |
| **LdMNPV *hr*8** | **1** |  | **158,292** | **8** | **4** | **14** | **1** | **5** | **41.4** | **25** | **34.4** | **100** | **21.4** | **158,371** |
| **SeMNPV *hr*2** | **1** | **AF169823.1** | **42,038** | **2** | **3** | **14** | **0** | **5** | **55.5** | **33.3** | **67.5** | **0** | **60.9** | **42,112** |
| **SeMNPV *hr*3** | **2** |  | **52,821** | **7** | **2** | **15** | **6** | **2** | **54.5** | **0** | **60** | **50** | **78.6** | **52,904** |
|  |  |  | **53,105** | **14** | **0** | **16** | **2** | **13** | **60** | **0** | **72.5** | **100** | **52.8** | **53,222** |
| **SeMNPV *hr*5** | **1** |  | **105,180** | **9** | **1** | **15** | **5** | **2** | **74.1** | **0** | **62.5** | **60** | **62.5** | **105,260** |
| **SeMNPV *hr*6** | **3** |  | **119,852** | **8** | **3** | **14** | **5** | **14** | **63.3** | **100** | **57.5** | **60** | **78** | **119,970** |
|  |  |  | **120,088** | **2** | **2** | **8** | **0** | **5** | **66.7** | **100** | **53.8** | **0** | **50** | **120,138** |
|  |  |  | **120,143** | **9** | **1** | **17** | **1** | **2** | **76** | **0** | **60** | **0** | **66.7** | **120,218** |
| **SeMNPV**  **non-*hr*** | **1** |  | **83,225** | **3** | **1** | **10** | **2** | **6** | **100** | **0** | **67.5** | **50** | **71.4** | **83,299** |
| **OpMNPV**  **non-*hr*** | **9** | **U75930.2** | **128,265** | **5** | **6** | **8** | **3** | **3** | **20** | **50** | **73.7** | **66.7** | **35.7** | **128,321** |
|  |  |  | **128,338** | **4** | **1** | **11** | **2** | **3** | **69.2** | **0** | **74.1** | **50** | **54.5** | **128,391** |
|  |  |  | **128,395** | **3** | **4** | **7** | **3** | **5** | **61.9** | **50** | **70.6** | **75** | **35.3** | **128,457** |
|  |  |  | **128,533** | **6** | **0** | **8** | **1** | **2** | **40** | **0** | **36** | **100** | **44.4** | **128,587** |
|  |  |  | **128,677** | **5** | **5** | **11** | **3** | **7** | **35.3** | **80** | **54.1** | **33.3** | **31.6** | **128,776** |
|  |  |  | **128,785** | **3** | **1** | **12** | **1** | **4** | **46.1** | **0** | **15.4** | **100** | **9.1** | **128,849** |
|  |  |  | **128,839** | **4** | **4** | **7** | **3** | **5** | **9.1** | **50** | **13.6** | **33.3** | **0** | **128,892** |
|  |  |  | **128,879** | **5** | **1** | **9** | **1** | **7** | **0** | **100** | **11.5** | **0** | **26.1** | **128,943** |
|  |  |  | **129,154** | **2** | **2** | **5** | **2** | **2** | **25** | **50** | **11.8** | **50** | **44.4** | **129,191** |
| **CrleGV non-*hr*** | **4** | **AY229987.1** | **20,625** | **7** | **1** | **8** | **4** | **2** | **63.2** | **100** | **38.5** | **50** | **36.4** | **20,685** |
|  |  |  | **20,761** | **5** | **5** | **5** | **2** | **3** | **50** | **60** | **57.1** | **50** | **41.7** | **20,814** |
|  |  |  | **21,451** | **2** | **2** | **10** | **3** | **4** | **50** | **50** | **45.4** | **33.3** | **50** | **21,512** |
|  |  |  | **21,567** | **6** | **2** | **12** | **2** | **2** | **66.7** | **50** | **55.9** | **50** | **33.3** | **21,634** |

The common structure found in all the baculovirus ORIs is described in this table, indicating the first and last nucleotide (nt) involved (refers as Start and End columns) and the length and AT content (%) of each component (S1: stem 1; Linker 1; S2: Stem 2; Linker 2; S3: Stem 3). The first column shows the name of the reported ORI, indicating the species to which it belongs. GB ID: GenBank Identifier.
