# Supplementary material for: Identification of Multiple Replication Stages and Origins in the Nucleopolyhedrovirus of Anticarsia gemmatalis
Source: Viruses. 2019 Jul 15;11(7):648. doi: 10.3390/v11070648 (PMC6669502; doi:10.3390/v11070648)
Supplement: Supplementary file 1 [file viruses-11-00648-s001.zip › Supplementary files/viruses-529304 supplementary.docx]

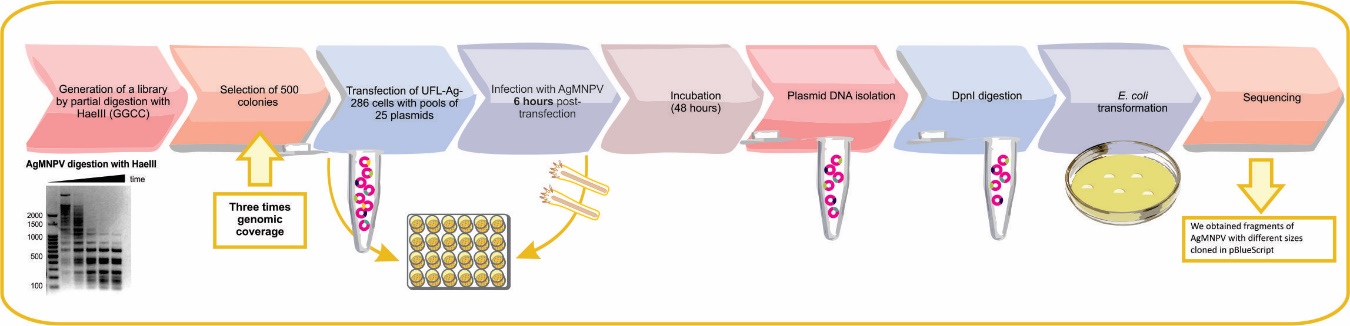


**Figure S1. Workflow to discover ORI sequences in AgMNPV.** The genome of AgMNPV was screened for DNA replication ORIs using an infection-dependent DNA replication assay in UFL-Ag-286 cells. For this, a genomic library of 500 constructs (produced by virus DNA partially fragmented with HaeIII and then molecularly cloned in *Escherichia coli* Dam+) were transfected in groups of 25. Input DNA (methylated on deoxyadenosine residues) was eliminated using DpnI and newly replicated DNA (non-methylated) was recovered by *E. coli* transformation. The identity of each insert was revealed by sequencing.


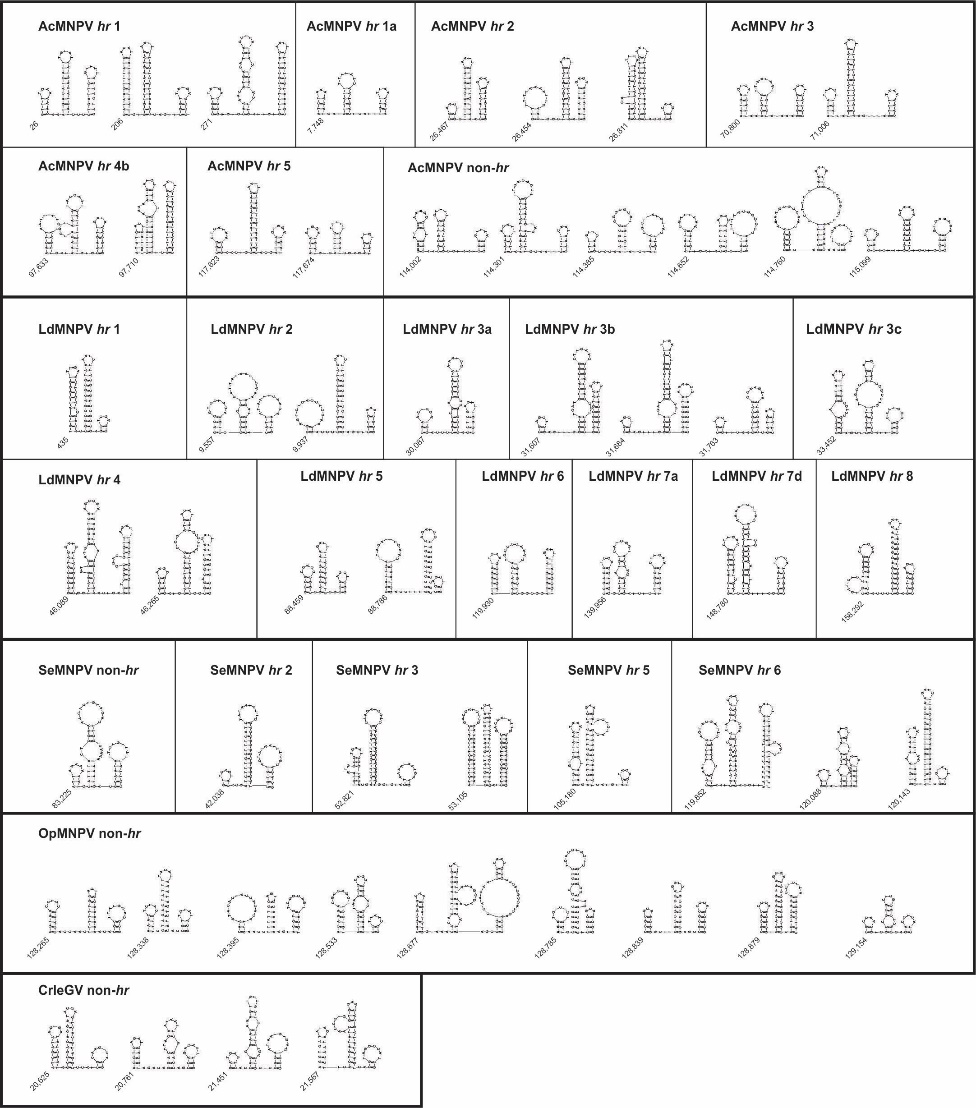


**Figure S2.** Baculovirus ORI sequences and their putative structure. The illustration shows the putative secondary structures of previously reported ORI in *Baculoviridae*. The nucleotide genome position of each sequence is indicated at the beginning of the DNA structure.

**Table S1.** Putative DNA structures of Baculovirus ORIs.

| **Name** | **Number of structures** | **GB ID** | **Start (nt)** | **Length** | | | | | **AT%** | | | | | **End (nt)** |
| --- | --- | --- | --- | --- | --- | --- | --- | --- | --- | --- | --- | --- | --- | --- |
|  |  |  |  | **S1 (bp)** | **Linker 1**  **(nt)** | **S2**  **(bp)** | **Linker 2**  **(nt)** | **S3**  **(bp)** | **S1** | **Linker 1** | **S2** | **Linker 2** | **S3** |  |
| **AcMNPV *hr*1** | **3** | **KM667940.1** | **26** | **4** | **2** | **9** | **3** | **7** | **75** | **100** | **74.1** | **0** | **66.7** | **90** |
|  |  |  | **206** | **9** | **2** | **11** | **5** | **4** | **64.3** | **50** | **60** | **60** | **38.5** | **283** |
|  |  |  | **271** | **4** | **4** | **10** | **5** | **12** | **38.5** | **50** | **63.6** | **80** | **60** | **355** |
| **AcMNPV *hr*1a** | **1** |  | **7,748** | **4** | **3** | **6** | **5** | **4** | **81.8** | **66.7** | **73.7** | **80** | **83.3** | **7865** |
| **AcMNPV *hr*2** | **3** |  | **26,467** | **2** | **1** | **11** | **1** | **6** | **37.5** | **0** | **73.1** | **100** | **66.7** | **26,519** |
|  |  |  | **26,454** | **3** | **4** | **11** | **1** | **5** | **82.3** | **25** | **73.1** | **100** | **55.5** | **26,519** |
|  |  |  | **26,811** | **10** | **0** | **12** | **3** | **2** | **71.4** | **0** | **60** | **25** | **28.6** | **26,876** |
| **AcMNPV *hr*3** | **2** |  | **70,800** | **5** | **2** | **5** | **5** | **5** | **71.4** | **100** | **72.2** | **100** | **50** | **70,852** |
|  |  |  | **71,006** | **4** | **2** | **13** | **6** | **4** | **76.9** | **100** | **68.7** | **83.3** | **83.3** | **71,070** |
| **AcMNPV *hr*4b** | **2** |  | **97,633** | **5** | **3** | **9** | **3** | **5** | **78.9** | **66.6** | **78.6** | **33.3** | **66.6** | **97,703** |
|  |  |  | **97,710** | **5** | **0** | **11** | **2** | **12** | **76.9** | **0** | **64.5** | **50** | **60** | **97,782** |
| **AcMNPV *hr*5** | **2** |  | **117,623** | **3** | **5** | **12** | **3** | **4** | **38.4** | **60** | **63.3** | **33.3** | **61.5** | **117,686** |
|  |  |  | **117,674** | **4** | **3** | **5** | **4** | **3** | **61.5** | **33.3** | **86.6** | **25** | **70** | **117,718** |
| **AcMNPV**  **non-*hr*** | **6** |  | **114,002** | **5** | **2** | **7** | **6** | **3** | **33.3** | **100** | **45** | **80** | **36.4** | **114,057** |
|  |  |  | **114,301** | **4** | **1** | **11** | **6** | **4** | **61.5** | **100** | **70.6** | **66.7** | **75** | **114,366** |
|  |  |  | **114,385** | **3** | **4** | **6** | **4** | **4** | **60** | **50** | **35** | **75** | **63.2** | **114,441** |
|  |  |  | **114,652** | **5** | **5** | **5** | **2** | **4** | **66.7** | **80** | **64.7** | **50** | **52.4** | **114,713** |
|  |  |  | **114,760** | **5** | **2** | **9** | **2** | **2** | **36.4** | **50** | **65.8** | **100** | **57.1** | **114,840** |
|  |  |  | **115,099** | **3** | **5** | **7** | **5** | **4** | **27.3** | **80** | **30** | **40** | **31.2** | **115,155** |
| **LdMNPV *hr*1** | **1** | **AF081810.1** | **435** | **11** | **1** | **13** | **1** | **2** | **51.8** | **0** | **34.4** | **100** | **25** | **503** |
| **LdMNPV *hr*2** | **2** |  | **9,557** | **4** | **1** | **6** | **1** | **4** | **37.5** | **0** | **55.2** | **100** | **78.9** | **9,622** |
|  |  |  | **9,937** | **2** | **4** | **13** | **4** | **4** | **38.9** | **25** | **28.1** | **50** | **27.3** | **10,005** |
| **LdMNPV *hr*3a** | **1** |  | **30,087** | **3** | **4** | **11** | **1** | **5** | **37.5** | **37.5** | **28.1** | **100** | **35.7** | **30,087** |
| **LdMNPV *hr*3b** | **3** |  | **31,607** | **2** | **6** | **12** | **0** | **9** | **25** | **66.7** | **42.1** | **0** | **45.4** | **31,680** |
|  |  |  | **31,684** | **2** | **6** | **12** | **0** | **8** | **25** | **66.7** | **47.5** | **0** | **40.9** | **31,759** |
|  |  |  | **31,763** | **2** | **5** | **7** | **1** | **4** | **25** | **80** | **38.1** | **0** | **54.5** | **31,808** |
| **LdMNPV *hr*3c** | **1** |  | **33,452** | **9** | **4** | **9** | **3** | **3** | **39.3** | **50** | **57.1** | **100** | **15.4** | **33,533** |
| **LdMNPV *hr*4** | **2** |  | **46,089** | **9** | **2** | **13** | **5** | **11** | **40.9** | **0** | **47.5** | **60** | **34.4** | **46,189** |
|  |  |  | **46,265** | **4** | **3** | **11** | **2** | **10** | **33.3** | **50** | **50** | **0** | **44** | **46,344** |
| **LdMNPV *hr*5** | **2** |  | **88,459** | **4** | **1** | **9** | **2** | **3** | **7.7** | **0** | **50** | **0** | **20** | **88,506** |
|  |  |  | **88,786** | **7** | **6** | **10** | **0** | **2** | **61.5** | **66.7** | **32.1** | **0** | **25** | **88,853** |
| **LdMNPV *hr*6** | **1** |  | **119,930** | **7** | **0** | **6** | **5** | **8** | **44.4** | **0** | **45.8** | **0** | **55** | **119,996** |
| **LdMNPV *hr*7a** | **1** |  | **139,956** | **6** | **1** | **7** | **5** | **6** | **25** | **0** | **54.2** | **60** | **50** | **140,019** |
| **LdMNPV *hr*7d** | **1** |  | **148,780** | **9** | **1** | **13** | **5** | **5** | **44** | **0** | **35** | **80** | **41.2** | **148,867** |
| **LdMNPV *hr*8** | **1** |  | **158,292** | **8** | **4** | **14** | **1** | **5** | **41.4** | **25** | **34.4** | **100** | **21.4** | **158,371** |
| **SeMNPV *hr*2** | **1** | **AF169823.1** | **42,038** | **2** | **3** | **14** | **0** | **5** | **55.5** | **33.3** | **67.5** | **0** | **60.9** | **42,112** |
| **SeMNPV *hr*3** | **2** |  | **52,821** | **7** | **2** | **15** | **6** | **2** | **54.5** | **0** | **60** | **50** | **78.6** | **52,904** |
|  |  |  | **53,105** | **14** | **0** | **16** | **2** | **13** | **60** | **0** | **72.5** | **100** | **52.8** | **53,222** |
| **SeMNPV *hr*5** | **1** |  | **105,180** | **9** | **1** | **15** | **5** | **2** | **74.1** | **0** | **62.5** | **60** | **62.5** | **105,260** |
| **SeMNPV *hr*6** | **3** |  | **119,852** | **8** | **3** | **14** | **5** | **14** | **63.3** | **100** | **57.5** | **60** | **78** | **119,970** |
|  |  |  | **120,088** | **2** | **2** | **8** | **0** | **5** | **66.7** | **100** | **53.8** | **0** | **50** | **120,138** |
|  |  |  | **120,143** | **9** | **1** | **17** | **1** | **2** | **76** | **0** | **60** | **0** | **66.7** | **120,218** |
| **SeMNPV**  **non-*hr*** | **1** |  | **83,225** | **3** | **1** | **10** | **2** | **6** | **100** | **0** | **67.5** | **50** | **71.4** | **83,299** |
| **OpMNPV**  **non-*hr*** | **9** | **U75930.2** | **128,265** | **5** | **6** | **8** | **3** | **3** | **20** | **50** | **73.7** | **66.7** | **35.7** | **128,321** |
|  |  |  | **128,338** | **4** | **1** | **11** | **2** | **3** | **69.2** | **0** | **74.1** | **50** | **54.5** | **128,391** |
|  |  |  | **128,395** | **3** | **4** | **7** | **3** | **5** | **61.9** | **50** | **70.6** | **75** | **35.3** | **128,457** |
|  |  |  | **128,533** | **6** | **0** | **8** | **1** | **2** | **40** | **0** | **36** | **100** | **44.4** | **128,587** |
|  |  |  | **128,677** | **5** | **5** | **11** | **3** | **7** | **35.3** | **80** | **54.1** | **33.3** | **31.6** | **128,776** |
|  |  |  | **128,785** | **3** | **1** | **12** | **1** | **4** | **46.1** | **0** | **15.4** | **100** | **9.1** | **128,849** |
|  |  |  | **128,839** | **4** | **4** | **7** | **3** | **5** | **9.1** | **50** | **13.6** | **33.3** | **0** | **128,892** |
|  |  |  | **128,879** | **5** | **1** | **9** | **1** | **7** | **0** | **100** | **11.5** | **0** | **26.1** | **128,943** |
|  |  |  | **129,154** | **2** | **2** | **5** | **2** | **2** | **25** | **50** | **11.8** | **50** | **44.4** | **129,191** |
| **CrleGV non-*hr*** | **4** | **AY229987.1** | **20,625** | **7** | **1** | **8** | **4** | **2** | **63.2** | **100** | **38.5** | **50** | **36.4** | **20,685** |
|  |  |  | **20,761** | **5** | **5** | **5** | **2** | **3** | **50** | **60** | **57.1** | **50** | **41.7** | **20,814** |
|  |  |  | **21,451** | **2** | **2** | **10** | **3** | **4** | **50** | **50** | **45.4** | **33.3** | **50** | **21,512** |
|  |  |  | **21,567** | **6** | **2** | **12** | **2** | **2** | **66.7** | **50** | **55.9** | **50** | **33.3** | **21,634** |

The common structure found in all the baculovirus ORIs is described in this table, indicating the first and last nucleotide (nt) involved (refers as Start and End columns) and the length and AT content (%) of each component (S1: stem 1; Linker 1; S2: Stem 2; Linker 2; S3: Stem 3). The first column shows the name of the reported ORI, indicating the species to which it belongs. GB ID: GenBank Identifier.
