# Supplementary figures and images for: Identification of Multiple Replication Stages and Origins in the Nucleopolyhedrovirus of Anticarsia gemmatalis
Source: Viruses. 2019 Jul 15;11(7):648. doi: 10.3390/v11070648 (PMC6669502; doi:10.3390/v11070648)

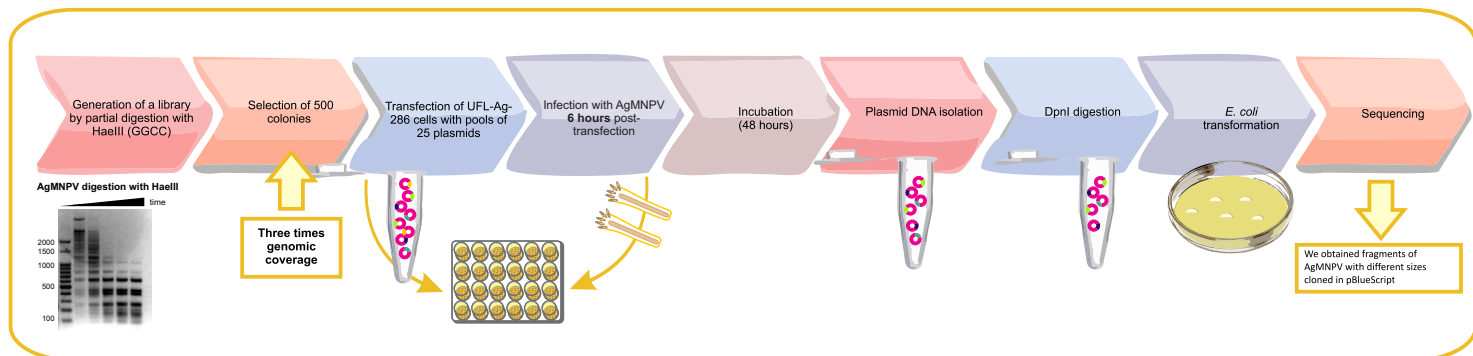

Supplement: Supplementary file 1 [file viruses-11-00648-s001.zip › Supplementary files/Figure S1.pdf]

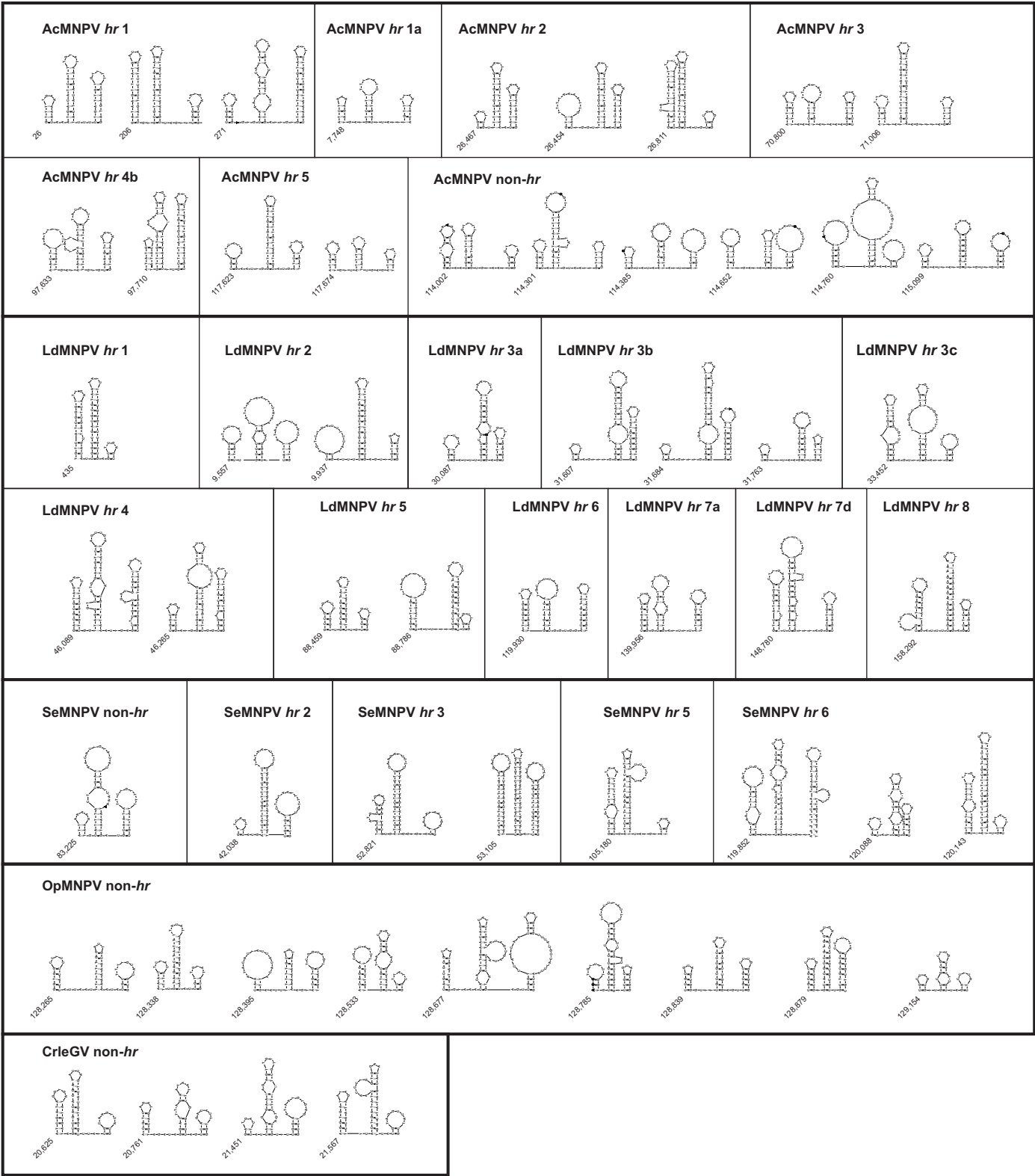

Supplement: Supplementary file 1 [file viruses-11-00648-s001.zip › Supplementary files/Figure S2.pdf]
